# Clearing Hospitals for the Territorial Medical Service

**Published:** 1909-09-25

**Authors:** 


### The Royal Army Medical Corps Section.

### CLEARING HOSPITALS FOR THE TERRITORIAL MEDICAL SERVICE.

Of the different duties that fall to the Royal Army Medical Corps that of the collection of the sick and wounded of the fighting force and their removal from the area of active operations is one of the most important as well as one of the greatest difficulty. Should an invasion of these islands take place and fighting ensue, it would be the removal of these casualties that would be the main function of the Territorial Medical Corps, and it is important to draw attention to a gap that exists in the territorial scheme of medical assistance. Before, however, doing so, it is only right to say that this incompleteness of the Territorial Medical Service was not an oversight, but was intentional, and was created with the object of allowing the Voluntary Aid Societies that exist in our midst—namely, the British Red Cross Society, the St. John's Ambulance Association, and the St. Andrew's Ambulance Association—to step in and supply the deficiency, thus giving the civilian element in the population an opportunity of helping in the defence of the country.

To understand this matter it must be remembered that the territorial field organisation for the removal of the sick and wounded from the whole area of active operations is modelled on that of the Regular Army, which is based on the principle of having three zones—(1) the "collecting," (2) the "evacuating," and (3) the "distributing." The "collecting" zone is situated in the battlefield and its vicinity, and the necessary assistance is rendered by the regimental medical officers, the regimental stretcher-bearers, and the field ambulances. These bodies work in unison and simultaneously they render first-aid treatment and convey the wounded to the rear. It is not necessary to describe here in detail how this is done, but at the end of a battle the result is that the wounded find themselves being cared for and treated by the field ambulances. These latter units are, however mobile bodies, and are organised to move with the troops, so that they must be ready to play the same *rôle* of assistance should another battle be fought the following day. This they cannot do if their tent divisions and ambulance wagons are filled with wounded. Consequently they must be relieved of their patients without any delay, so as to leave them free to move on with the Army should an advance be made. In the Regular Army this is done by a special unit, the clearing hospital, which takes over the wounded from the field ambulances and makes all necessary arrangements for their further transference, if necessary, to the rear. These clearing hospitals represent the second or "evacuating" zone, and their special function is to receive the wounded and care for them until they can be evacuated by road, rail, or water, and transported to the next or "distributing" zone, with its general hospitals and convalescent homes at the base. The proportion of clearing hospitals allowed in the Regular Army is one for each division, and it is formed whenever the division is mobilised. Each clearing hospital contains 200 beds, and it is staffed in the same proportion as a stationary hospital, so that its *personnel* will consist of eight medical officers, one quartermaster, and eighty rank and file, but there will be no Army nurses of the Queen Alexandra Imperial Military Nursing Service attached to it. As regards its location, this must vary with circumstances, but as far as possible it will be placed so as to be available for use at the head of the lines of communication, and be within the reach of the field ambulances so as to relieve the latter of their sick and wounded and allow them to follow up the Army.

This maintenance of connection with the field ambulances is the main business of a clearing hospital, and there may be circumstances where it may be necessary not only to keep in touch with them by sending forward a small proportion of the *personnel*, but even, as in the case of a rapid advance of the Army, for the whole clearing hospital to go right up to the field ambulances and take over their sick and wounded on the spot. Having thus relieved the field ambulances of their sick and wounded, the clearing hospital must at once turn its attention to getting rid of its occupants, for it is designed to give only temporary shelter to them, and is merely the channel by which they are passed on to the stationary hospitals erected on the lines of communication or to the general hospitals. In fact, a clearing hospital may serve as a centre for sorting out the patients. In the case of a battle fought in our own country during an attempted invasion, those not seriously hurt, yet not able to rejoin their regiments for some time, might be sent to their own homes, while those severely hurt might receive any further necessary treatment and then be transmitted at once to the rear or detained for a few days. This latter course might also be followed in connection with trivial cases that needed only two or three days' treatment to fit them to return to the ranks. Under no circumstances, however, must the clearing hospital become congested. It belongs to the "evacuating zone," and it should always be able to receive patients from the front. A very important factor in this work of evacuation is suitable transport for conveying the sick and wounded from the field ambulances to the clearing hospitals, and from there to the railway or any stationary hospital in the rear. In the Regular Army the arranging for this transport is one of the duties of the director of transport, and it is met by making use of the empty wagons of supply columns and parks returning to replenish at the advanced base for stores. If required, additional local transport is also provided by hire or requisition.

From what has been said it must be quite apparent that clearing hospitals play a very important part in the organisation for the removal of the casualties in war and their conveyance to a suitable place for their necessary treatment. In fact, they are really "the pivot upon which the whole system of evacuating sick and wounded turns." Representing as they do on a larger scale the tent divisions of the field ambulances, they form the central point upon which the "collecting zone" converges and from which the "evacuating" and "distributing" zones diverge. Closely associated with them are the matter of transport and several other duties that are inseparable from the removal of injured persons for any distance, such as the formation of "rest stations," where they may receive refreshment and be made comfortable while waiting to be loaded on trains or after they are taken off trains. These clearing hospitals do not as yet exist in the Territorial Medical Service, and as such assistance requires forethought and preparation, the military authorities are very properly turning their attention to supply this want in the scheme of territorial medical assistance. At present the indications are that they propose to include the various Voluntary Aid Organisations in the work of home defence and utilise their services for doing so.

